# The Seventieth Anniversary of the Pharmaceutical Society

**Published:** 1911-05-20

**Authors:** 


### THE SEVENTIETH ANNIVERSARY OF THE PHARMACEUTICAL SOCIETY.

The seventieth anniversary of the Pharmaceutical Society, which is being celebrated this week, is an event not altogether without interest to medical practitioners, for the Society, by its efforts to promote the advancement of pharmacy, has contributed in no small measure to the progress of medical science. A Bill to amend the laws relating to medical practice was introduced in the House of Commons in 1841 by a Mr. Hawes, by which it was proposed to make it an offence subject to a penalty of £20 to recommend any medicine for gain. A meeting of chemists and druggists was held in February 1841, and so powerful was the opposition they were able to make that Mr. Hawes was prevailed upon to omit all the clauses affecting chemists and druggists.

### The Foundation of the Society.

Encouraged by their success the chemists conceived the idea of creating a permanent organisation, and a meeting was held on April 15 of the same year, at the Crown and Anchor Tavern, when, on the motion of William Allen, F.R.S., it was unanimously resolved: "For the purpose of protecting the permanent interests and increasing the respectability of chemists and druggists, an association be now formed under the title of the Pharmaceutical Society of Great Britain." On June 1 of the same year the first meeting of the newly constituted Society was held, and William Allen was elected president. Within a few months a house was taken at 17 Bloomsbury Square, and the founders set to work to institute a system of education. Before the close of 1841 a school of pharmacy was founded and the services of some distinguished scientists secured as lecturers. A Preliminary examination for apprentices, to be passed before the execution of their indentures, a Minor examination for associates, and a Major examination for members were established. A museum and a library were also founded, both of which have grown to important dimensions. Two years after the Society had been founded it received its Royal Charter of Incorporation. The obtaining of this Charter was an extremely important event, for it gave the Society a standing among scientific and learned bodies. At that time the practice of pharmacy, the use of titles, and the sale of poisons were unrestricted by law. Early in its history, therefore, the Society turned its attention to the question of legislation. In 1852 a Bill was introduced by which it was proposed to prevent unqualified persons from carrying on the business of pharmaceutical chemist, and to protect certain titles. Apparently the time was not ripe for the demand to be accorded in full, and the Bill, as finally passed, made it an offence for unqualified persons to use the title of pharmaceutical chemist, etc., but did not restrict the practice of pharmacy.

### Later Legislation.

From 1857 onwards Bills were introduced to regulate the sale of poisons, and towards the end of 1858 the public desire for some regulation was stimulated by an alarming accident that occurred at Bradford. A lozenge manufacturer was supplied with arsenic instead of sulphate of lime by a druggist's assistant, and as a result of the mistake twenty people died and two hundred became more or less seriously ill. A Bill drafted immediately afterwards was abandoned, however, and the same fate was meted out to several other Bills until 1868, when the second Pharmacy Act, and the one which mainly regulates the sale of poisons at the present time, was passed. This protected the title of "chemist and druggist," and restricted to registered persons the sale of certain poisons. Twelve years later, however, it was shown by a judicial decision of the House of Lords that although it was an offence for an unqualified person to sell poisons and do certain other things, it was not an offence for a company of unqualified persons to do these things. This decision gave an impetus to the company movement, which grew to threatening dimensions, and in course of time endangered the privileged position of chemists and druggists and established competition, the effect of which was, in many cases, disastrous to individuals and baneful to the whole calling of pharmacy. In spite of constant endeavours to remove the anomaly of the law which allowed this state of affairs, the Pharmaceutical Society was unsuccessful, although by the Act of 1908 companies were brought under the influence of the Pharmacy Act, but allowed to carry on business. More recently the activities of the Society have been mainly directed towards the improvement of its organisation and the betterment of the existing educational system.

